# Cold agglutinin syndrome in a patient with metastatic breast cancer: a Case Report

**DOI:** 10.3389/fmed.2025.1711809

**Published:** 2026-01-16

**Authors:** Daniel Bandarra, Dina Rochate, Beatriz Gosalbez, José Ferreira, Nidia Maltez Cunha, Sara Carvalhal

**Affiliations:** 1 Department of Medical Oncology, ULS do Algarve, Faro, Portugal; 2 Department of Medical Hematology, ULS do Algarve, Faro, Portugal; 3 Algarve Biomedical Center, Research institute (ABC-Ri), Universidade do Algarve, Faro, Portugal

**Keywords:** breast cancer, chemotherapy, cold agglutinin syndrome (CAS), autoimmune hemolytic anemia (AIHA), drug-induced hemolysis

## Abstract

**Background:**

Cold agglutinin syndrome (CAS) is a form of autoimmune hemolytic anemia (AIHA), most often associated with lymphoproliferative disorders or infections. Its occurrence in breast cancer is rare and may be triggered by systemic treatment.

**Case presentation:**

We report the case of a woman in their fifties diagnosed with breast cancer in 2019. She underwent surgery followed by adjuvant chemotherapy and radiotherapy and subsequently received 3 years of endocrine therapy before developing bone and hepatic metastases. First-line treatment with ribociclib plus letrozole achieved partial response, and fulvestrant was administered at progression. Following further progression, paclitaxel was introduced as third-line metastatic therapy. After four weekly administrations, the patient was admitted to our hospital with severe anemia and diagnosed with CAS. Prompt management and a multidisciplinary approach resulted in partial hematological recovery. Nevertheless, paclitaxel was permanently discontinued, and subsequent therapies provided only transient benefit. The disease continued to progress, her performance status declined, and she ultimately transitioned to exclusive palliative care until death.

**Conclusion:**

This case illustrates a rare and severe immune complication of paclitaxel in metastatic breast cancer. The emergence of CAS not only limited systemic options but also reshaped the therapeutic trajectory, highlighting the need for close monitoring during cancer treatments. Early recognition, multidisciplinary approach, and prompt management can provide some improvement, although overall prognosis remains determined by the underlying malignancy.

## Introduction

Breast cancer is the most common malignancy among women and a leading cause of cancer-related mortality worldwide (1). In the metastatic setting, anemia is a frequent complication, most often resulting from treatment-induced myelosuppression, nutritional deficiencies, or bone marrow infiltration (2). Less commonly, anemia may result from an autoimmune mechanism such as autoimmune hemolytic anemia (AIHA). AIHA is a rare form of hemolytic anemia caused by autoantibodies that target red blood cells, often in a temperature-dependent manner. It encompasses cold AIHA, which is further classified into primary cold agglutinin disease (CAD), paroxysmal cold hemoglobinuria (PCH), and secondary cold agglutinin syndrome (CAS) (3–5). Cold AIHA is rarely reported in association with solid tumors (6–8).

While cold AIHA can occasionally arise as a paraneoplastic manifestation, the association with systemic therapy raises the possibility of a drug-induced mechanism (9). In such cases, the drug may trigger the formation of antibodies that recognize red blood cell surface antigens and, at low temperatures, activate the classical complement pathway. These antibodies can bind to erythrocytes, promote complement deposition, and ultimately cause intravascular or extravascular hemolysis (10).

Among anticancer treatments, taxane-based regimens have occasionally been linked to immune-mediated hemolysis (9, 11). Awareness of this potential complication is crucial, as timely recognition can directly shape therapeutic decision-making, often requiring discontinuation of the causative agent, adjustment of systemic treatment, and the need to prioritize multidisciplinary care. Beyond altering the oncological trajectory, it also carries major implications for prognosis.

Here, we report the development of CAS in a patient with metastatic breast cancer following taxane-based chemotherapy.

## Case presentation

**FIGURE 1:**
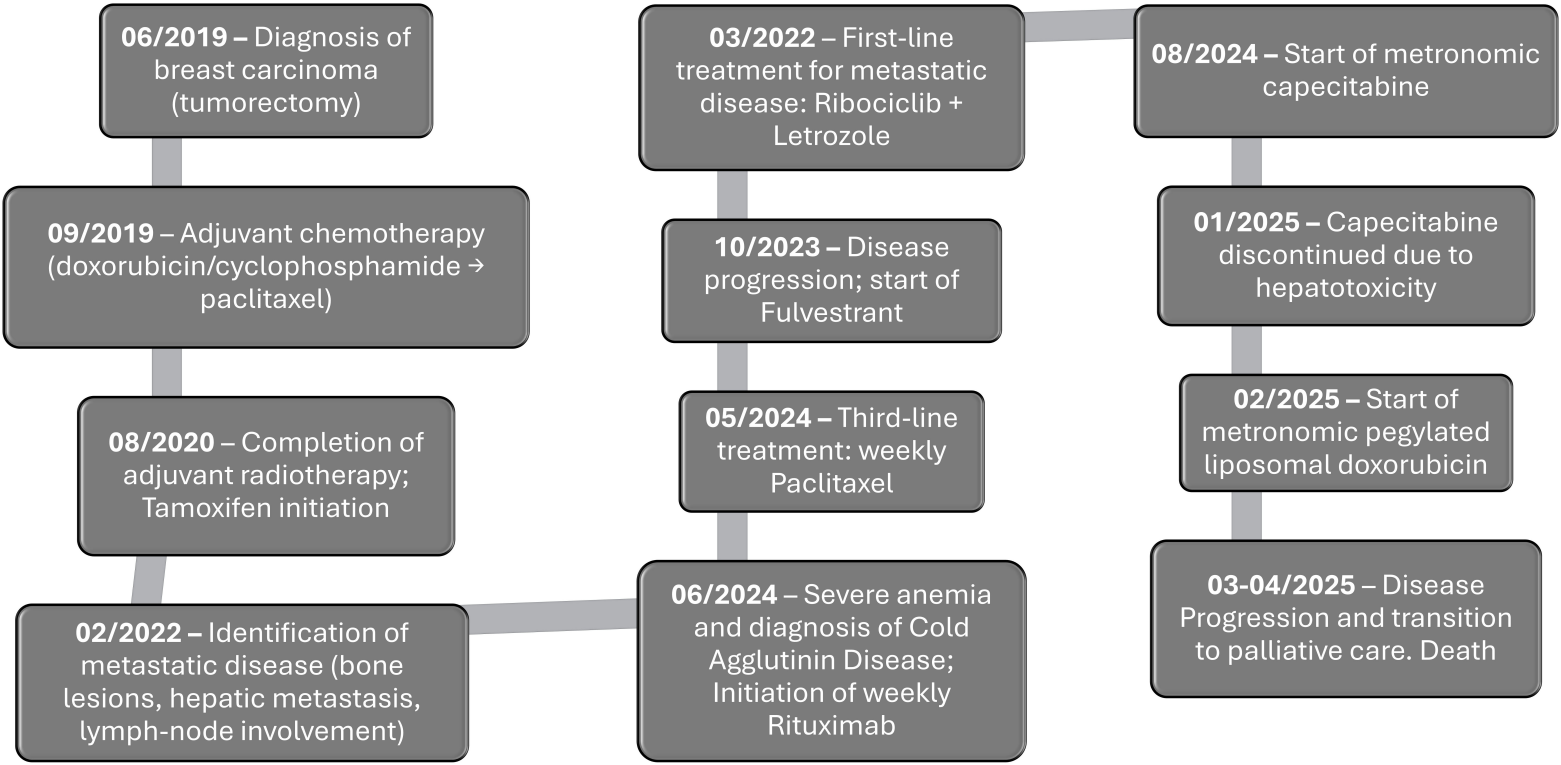
Clinical timeline of the patient with metastatic breast carcinoma and CAS. The diagram summarizes key milestones from initial breast cancer diagnosis through adjuvant therapy, detection of metastatic disease, sequential systemic treatments, onset and management of CAS, and final transition to palliative care and death. Dates (month/year) indicate the start of each major event or intervention.

A woman in the 50–59 age range with a medical history of obesity, hypertension, dyslipidemia, and anxiety was diagnosed in 2019 with left breast carcinoma. Histopathology revealed invasive carcinoma of no special type, grade 2, estrogen receptor (ER) positive, progesterone receptor (PR) positive, and HER2-negative. She underwent tumorectomy followed by adjuvant chemotherapy with doxorubicin/cyclophosphamide, followed by paclitaxel (Figure 1). Treatment was complicated by mild toxicities, including nausea, vomiting, asthenia, and peripheral neuropathy. Radiotherapy was completed in August 2020, and tamoxifen was initiated.

In February 2022, PET-CT demonstrated diffuse bone metastases, a single hepatic lesion, and peripancreatic lymphadenopathy. Liver biopsy confirmed metastatic breast carcinoma (ER/PR positive, HER2-negative). After the diagnosis of metastatic disease, ribociclib plus letrozole was initiated as first-line metastatic treatment, achieving partial response. Following further progression, the patient received fulvestrant and subsequently paclitaxel as second- and third-line therapies, respectively, with the latter initiated in May 2024 (Figure 1). After four weekly paclitaxel treatments (80 mg/m^2^/week), the patient was admitted to our hospital with severe anemia (Hb 5.5 g/dL, in-hospital nadir 2.8 g/dL). Laboratory evaluation revealed evidence of hemolysis, including elevated total bilirubin (3.1 mg/dL, nadir of 5.5 mg/dL), elevated lactacte dehydrogenase (1107 UI/L), low haptoglobin (<7 mg/dL), and a reticulocyte count of 7.3% (32 × 10^9^/L; reference range 50–100 × 10^9^/L). It was also observed a direct antiglobulin test (DAT) strongly positive (3+), with C3d 4+ and IgM 2+ (Table 1). The peripheral smear showed marked red blood cell agglutination, polychromasia, scarce spherocytes and no schistocytes were identified. The slide was rewarmed to 37 °C before preparation and red blood cell agglutination was dispersed. A comprehensive infectious and autoimmune workup was conducted, yielding negative results.

**TABLE 1:** Evolution of hematologic and serologic findings during the patient’s admission, nadir and discharge.

Parameter	At admission	Nadir/peak during hospitalization	At discharge	Reference range
Hemoglobin (g/L)	5.5	2.8	8.6	12–16
Total bilirubin (mg/dL)	3.1	5.5	0.4	0.2–1.2
Lactate dehydrogenase (U/L)	1107	–	340	125–243
Haptoglobin (mg/dL)	<7	–	–	30–200
DAT (polyspecific)	3+			Negative
DAT (C3d)	4+			Negative
DAT (IgM)	2+			Negative

The direct antiglobulin test (DAT) was performed using the test tube (polyspecific) method, followed by confirmation using a CARD system (monospecific, C3d, IgM).

The patient required multiple life-saving transfusions administered through a blood warmer, and corticosteroids for 6 days (prednisolone, 1 mg/Kg/day, tapering 7.5 mg/week) with a limited response. Due to the patient’s clinical instability at the time of hemoglobin aggravation and apparent disease progression, bone marrow infiltration could not be assessed. Hepatitis serology was negative. Rituximab 375 mg/m^2^ weekly for four consecutive doses was subsequently initiated, resulting in clinical and hematological improvement, with hemoglobin rising to 7–8 g/dL, and hemolysis parameters decreased.

Following discharge, paclitaxel was not resumed. A few weeks later, the patient started metronomic capecitabine (500 mg twice daily), initially well tolerated but discontinued in January 2025 due to hepatotoxicity (AST/ALT > 5 × ULN, bilirubin 1.7 mg/dL), and worsening clinical condition. In February 2025, she initiated metronomic liposomal doxorubicin (20 mg/m^2^ every 2 weeks), which produced transient biochemical improvement, including reduced AST, normalized bilirubin, and decreased CA15-3. However, by March 2025, PET-CT revealed diffuse hepatic and skeletal progression. Systemic therapy was permanently discontinued, and she transitioned to exclusive best supportive and palliative care until her death (Figure 1).

## Discussion

Cold agglutinin disease is a subtype of AIHA mediated by cold agglutinins-IgM autoantibodies capable of agglutinating red blood cells by binding to the surface antigen. Primary CAD is characterized by chronic hemolysis, a significant cold agglutinin titer, typical DAT findings, and the absence of an underlying clinical disorder (3–5, 12). The characteristic DAT pattern is a positive monospecific test for C3d only. However, DAT results may be weakly positive for IgG in addition to C3d in up to 20% of patients. Determination of the thermal amplitude is a time-consuming procedure and, in most cases, unnecessary for establishing a reliable diagnosis. Nevertheless, it can be helpful in selected cases to distinguish pathologic cold agglutinins from physiological low-titer cold agglutinins that may cause false-positive findings (3–5, 12).

Secondary CAS is even less common than CAD. In most patients, the onset of hemolysis is abrupt, presenting with pallor, jaundice, and occasionally prostration. In addition to biochemical evidence of hemolysis, high-titer cold agglutinins are typically detectable, and the direct antiglobulin test is positive for C3d (4, 5, 12).

In most patients with metastatic malignancy, anemia is often attributable to marrow suppression, nutritional deficiencies, or disease infiltration (13). The abrupt onset of severe hemolysis, shortly after paclitaxel initiation, supports the hypothesis of drug-induced immune hemolytic anemia, rather than a paraneoplastic mechanism. This interpretation is consistent with published cases in which taxane-based regimens and other anti-cancer drugs have been associated with immune-mediated hemolysis (9, 11, 14).

Several mechanisms may explain this complication: (i) paraneoplastic immune activation in the context of progressive disease; (ii) hepatic dysfunction from metastases further compromising immune tolerance and red blood cell clearance, and (iii) drug-induced immune dysregulation, whereby paclitaxel could have triggered cold-reactive IgM antibodies and complement activation, generating a drug resistance complication.

Management of CAD and CAS varies according to whether an underlying trigger is present. In secondary CAS, therapy is primarily directed at treating the underlying condition, when identifiable. Supportive measures such as red blood cell transfusion may be required, following the same precautions applied in primary CAD (4, 15, 16). Although measures such as corticosteroids have been described in case reports, their benefit remains poorly documented, especially since hemolysis often resolves when the underlying infection or disorder is treated (4). Emerging evidence supports the use of rituximab in selected cases of CAS when an underlying B-cell clone is identified; although data are limited, rituximab’s B-cell–depleting effect provides a mechanistic rationale for its use (17).

In primary CAD, corticosteroids are not recommended as first-line therapy due to limited and transient responses (<15%) (4, 18). Rituximab is the preferred treatment for patients requiring therapy, achieving response rates of approximately 50%–60% in clinical studies, with more durable remissions. It may be used alone or combined with agents such as bendamustine in refractory cases (13).

Despite partial hematological recovery, the onset of CAS in this patient had a profound impact on the oncological management. Paclitaxel was discontinued, and subsequent chemotherapy regimens had to be adapted to less myelosuppressive metronomic settings. Ultimately, recurrent hepatic dysfunction, cumulative toxicity, and functional decline restricted systemic options and accelerated transition to exclusive palliative care.

This case highlights how an uncommon, treatment-related immune complication can redefine the therapeutic trajectory of a patient with advanced breast cancer. By shifting the focus from disease progression to treatment toxicity, it illustrates the importance of recognizing unusual patterns of anemia, integrating hematology expertise, and adapting systemic strategies accordingly. Ultimately, it reminds us that oncology care is complex, and unexpected events can alter not only prognosis but also the very framework of decision-making.

## Conclusion

Cold agglutinin syndrome represents a rare and debilitating complication in the metastatic breast cancer setting. In this patient, its abrupt emergence shortly after paclitaxel highlights the need to consider treatment-related immune mechanisms alongside paraneoplastic causes. Beyond its rarity, the condition reshaped therapeutic priorities: systemic therapy was suspended, rituximab was introduced as salvage, and oncological decisions became driven as much by toxicity as by tumor progression. This case underlies how unexpected chemotherapy-induced complications can decisively alter management pathways, limiting therapeutic intensity, and hastening transition to supportive care. Close clinical monitoring and multidisciplinary involvement are key to recognizing and addressing such events, even in regions where cold agglutinin disease would not typically be anticipated.

## Data Availability

The raw data supporting the conclusions of this article will be made available by the authors, without undue reservation.
